# Delayed presentation of placenta accreta following a first‐trimester medical abortion

**DOI:** 10.1002/ccr3.7849

**Published:** 2023-08-25

**Authors:** Jenna Maurer, Sangeeta Ramani, Bo Xu, Stephen Gallousis, Mitchell Clark, Vaagn Andikyan

**Affiliations:** ^1^ Department of Obstetrics and Gynecology Stamford Hospital Stamford Connecticut USA; ^2^ Department of Pathology Stamford Hospital Stamford Connecticut USA; ^3^ Division of Gynecologic Oncology, Department of Obstetrics, Gynecology and Reproductive Sciences Yale School of Medicine New Haven Connecticut USA

**Keywords:** abnormal uterine bleeding, medical abortion, placenta accreta, uterine mass

## Abstract

Placenta accreta can rarely present as a uterine mass on imaging months after a first trimester medical abortion, even in patients at low‐risk for abnormal placentation. Early and accurate diagnosis can be crucial to reduce morbidity and mortality associated with this disease, particularly for those desiring fertility preservation.

## Graphical abstract

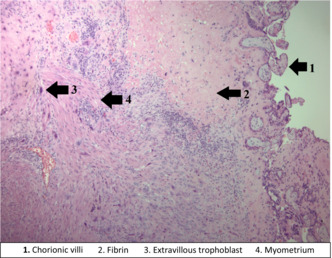

## 1 INTRODUCTION

Placenta accreta (PA) is a spectrum of disorders defined by abnormal adhesion of the placenta to the uterine myometrium. It can be further classified into accreta, increta, and percreta.^1^ The diagnosis of PA is typically made via ultrasound findings in the second or third trimester but can present clinically after a fetal delivery complicated by difficult placental removal and significant bleeding.^1^, ^2^ Rare cases of PA after first‐trimester dilation and curettage (D&C) have been reported, typically presenting with vaginal bleeding and occasionally with findings of uterine mass on ultrasonography and/or magnetic resonance imaging (MRI). On imaging, such findings can appear radiographically similar to several conditions including gestational trophoblastic disease or choriocarcinoma, uterine arteriovenous fistula, or degenerating fibroids.^3^ As such, diagnosis of PA after first‐trimester abortion proves to be both clinically and radiographically challenging.

Of the cases of PA after first‐trimester abortion that have been reported, most patients possess significant risk factors for abnormal placentation such as prior endometrial trauma in the form of cesarean section or D&C, or advanced maternal age (AMA).^4^ Based on a review article by Wang^4^ and colleagues investigating first‐trimester postabortion PA, up to 87% of the patients included in the study had a history of prior cesarean section. The same study shows that the majority of patients present within the first week following first‐trimester surgical abortion. Furthermore, the most common and definitive treatment for this condition is hysterectomy, reflecting the high risk of hemorrhage with conservative therapies such as uterine artery embolization.

This report describes a rare incidence of PA presenting with abnormal bleeding and a uterine mass on MRI, months after a first‐trimester medical abortion in a patient with minimal risk factors for abnormal placentation.

## 2 CASE

A 34‐year‐old gravida 6 para 2 with history of 2 spontaneous vaginal deliveries, 3 spontaneous abortions, and 1 medical termination of pregnancy presented with complaints of irregular menses. She presented to her primary OB/GYN in August, approximately 4 months after undergoing a first‐trimester medical termination in March–April 2022. She had no periods from April to July 2022, then resumed menstruation in August 2022. She took a pregnancy test at this time which was noted to be positive.

A pelvic ultrasound performed in September 2022 revealed an abnormal‐appearing endometrium with thickness of 24.07 mm and hyperemic blood flow. Subsequent pelvic MRI revealed a 4‐cm ill‐defined enhancing mass within the endometrial cavity, with multiple dilated vessels and suspected adjacent myometrial invasion. Preoperative serum B‐human chorionic gonadotrophin (b‐hCG) was 6425.0 mIU/mL, and chest X‐ray was unremarkable. With these findings, the patient was referred to our hospital for evaluation by gynecologic oncology. Differential diagnosis at this time included retained products of conception versus gestational trophoblastic disease. She was counseled on surgical options including D&C versus hysterectomy. She elected to proceed with hysterectomy due to high bleeding risk with D&C and no desire for future pregnancy.

**FIGURE 1:**
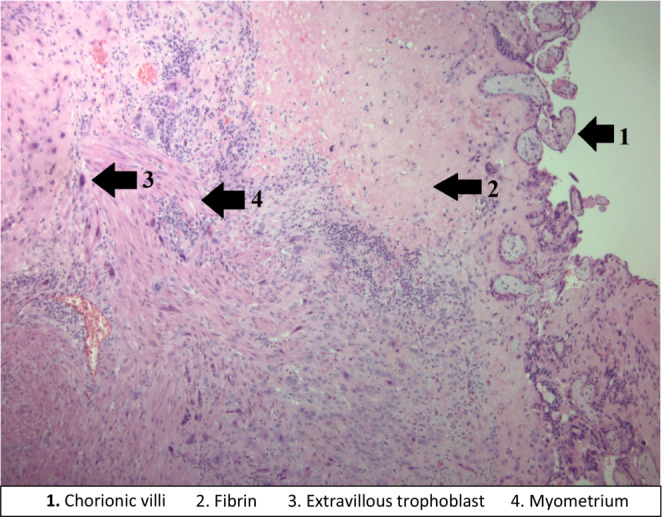
Placenta accreta. Chorionic villi superficially invade the myometrium without intervening decidua.

A laparoscopic hysterectomy and bilateral salpingectomy were performed. Intraoperatively, the uterus appeared to be vascular and boggy, approximately 8–10 weeks in size. The procedure was overall uncomplicated with an estimated blood loss of 100 cc. Pathologic analysis of the specimen identified a 4.2 × 3.9 × 1.5‐cm tan, red, ill‐defined friable mass on the anterior wall appearing to extend into the endometrium with up to 50% myometrial invasion. Final pathology showed a uterus consistent with PA without evidence of malignancy (Figure 1). Her postoperative course was unremarkable, and she was discharged home the same day. She was seen in the office for her postoperative visit and was found to be recovering well without complaints.

## 3 DISCUSSION

Placenta accreta is a spectrum of disease in which placental villi abnormally invade into uterine myometrium, with disease severity varying by depth of invasion.^5^ The most common presentation of PA appears immediately after delivery of the fetus, requiring either placental removal or hysterectomy. Massive obstetric hemorrhage frequently results and necessitates transfusion of blood products.^2^, ^5^ The incidence of PA has increased over recent years, which aligns with the increasing incidence of cesarean sections.^6^, ^7^ Other significant risk factors include placenta previa, PA in a prior pregnancy, AMA, and multiparity.^1^, ^6^

There are very few cases in the literature of accreta after a first‐trimester abortion. More commonly, they have been reported after the delivery of a full‐term fetus. The majority of cases in the literature of accreta after a first‐trimester abortion have presented with abnormal vaginal bleeding, with a fraction of these also presenting with a uterine mass on imaging.^3^, ^8^, ^9^ Most abnormal bleeding in such patients begins within a week of the procedure, with delayed presentation ranging from 1 week to 2 years in approximately 35% of cases.^4^ Furthermore, the majority of such cases have occurred after a first‐trimester D&C, not after a medical abortion, and in patients with at least one prior cesarean section.^5^

The case described in this report is exceedingly rare in that it occurred following a medical first‐trimester abortion, in a patient with no risk factors for PA except for multiparity. While PA is more readily considered for a patient with significant postpartum bleeding and a history of prior cesareans, this case highlights the clinical importance of increasing awareness of PA in a low‐risk patient. Prompt diagnosis is crucial for reducing morbidity and mortality associated with massive hemorrhage, especially in cases such as this that exhibit a delayed presentation of bleeding.

In summary, this case describes a unique presentation of PA as a uterine mass following a first‐trimester medical abortion, in a low‐risk patient with a delayed presentation of bleeding. To improve outcomes and decrease associated morbidity and mortality, clinicians must increase their clinical suspicion of PA outside of its typical presentation.

## AUTHOR CONTRIBUTIONS

**Jenna Maurer:** Conceptualization; investigation; visualization; writing – original draft; writing – review and editing. **Sangeeta Ramani:** Conceptualization; investigation; visualization; writing – original draft; writing – review and editing. **Bo Xu:** Investigation; visualization. **Stephen Gallousis:** Writing – review and editing. **Mitchell Clark:** Writing – review and editing. **Vaagn Andikyan:** Conceptualization; supervision; visualization; writing – review and editing.

## FUNDING INFORMATION

There is no funding to report for this research.

## CONFLICT OF INTEREST STATEMENT

The authors report no conflict of interest.

## CONSENT

Written informed consent was obtained from the patient to publish this report in accordance with the journal's patient consent policy.

## Data Availability

The data that support the findings of this study are available upon request from the corresponding author, JM. The data cannot be made publicly available due to institutional scope of practice policies.
